# Can leader self-sacrificial behavior inhibit unethical pro-family behavior? A personal identification perspective

**DOI:** 10.3389/fpsyg.2022.1078122

**Published:** 2023-01-12

**Authors:** Changchang Hou, Ken Cheng, Jiaying He, Panpan Hu, Yinghui Lin

**Affiliations:** ^1^School of Management, Zhejiang University of Technology, Hangzhou, China; ^2^School of Management, Shanghai University, Shanghai, China

**Keywords:** identification with the leader, leader self-sacrificial behavior, perceived insider status, personal identification, unethical pro-family behavior

## Abstract

As a kind of deviant and unethical behavior in the workplace, unethical pro-family behavior (UPFB) has recently received increased attention. Yet, the question of how to reduce UPFB remains less well understood. From the personal identification perspective, we hypothesize that leader self-sacrificial behavior (LSSB) inhibits employees’ UPFB through the mediation of identification with the leader. We further argue that employees’ perceived insider status enhances this hypothesized relationship. Our analysis of two-wave data collected from 236 Chinese employees indicated that identification with the leader partially mediated the negative relationship between LSSB and UPFB. Moreover, the effect of LSSB on identification with the leader and the aforementioned mediating relationship were stronger for employees who perceived themselves as insiders than outsiders. These findings provide theoretical implications for research on UPFB and LSSB and offer some suggestions that managers can follow to inhibit UPFB. Limitations and future research directions are also discussed.

## Introduction

Influenced by the COVID-19 pandemic and the global economic downturn, people are experiencing increased life difficulties (e.g., the unemployment of family members and the decrease in the family income). To alleviate these difficulties, more and more employees engage in pro-family acts in the workplace, even if these acts may likely cause damage to their organizations. For instance, a recent report showed that two employees embezzled nearly five thousand dollars from the company to fix up the residence they and their family lived in (Smith, 2020). For another example, a supermarket employee was charged with stealing goods to cut down household costs and feed the family (Chen, 2021). These acts are called unethical pro-family behavior (UPFB; Liu et al., 2020) and have gained increased attention in recent years (e.g., Cheng et al., 2022; Li et al., 2022; Yao et al., 2022).

UPFB refers to “an employee’s actions that are aimed at benefiting his or her entire family or specific family members, but which violate societal and organizational moral rules, norms, standards, laws, or codes” (Liu et al., 2020, p. 639), such as submitting household receipts to the company for reimbursement and taking company assets home for family use. By definition, UPFB is one kind of destructive deviant behavior that may likely hurt organizational interests (Liu et al., 2020). Thus, it is important for organizations to find effective ways to inhibit employees’ UPFB. Currently, there have existed two research streams to address this issue. One is to investigate the formation mechanisms of UPFB and appeal organizations to restrain the inducers of UPFB (e.g., family financial pressure; Liu et al., 2020; work–family conflict; Li et al., 2022). The other is to explore the inhibiting mechanisms of UPFB and recommend organizations to facilitate its inhibitors (e.g., family-supportive supervisor behavior; Cheng et al., 2022). Considering that strengthening inhibitors may be more practicable than weakening inducers and that leader behaviors usually have great impacts on employees, this study is to explore the inhibiting mechanism of employees’ UPFB from the lens of leader behaviors.

UPFB is conducted by employees to enhance family interests but is at the cost of organizational interests (Liu et al., 2020). Enlightened by this feature of UPFB, we deem that the question of how to inhibit UPFB may be addressed by two approaches, namely taking measures to safeguard employees’ family interests and to improve their emphasis on the organizational interests. In terms of the former approach, research has found that leaders’ family-supportive behaviors contributed to the fulfillment of employees’ family needs and thereby suppressed the occurrence of UPFB (Cheng et al., 2022). In contrast, little attention has been paid to the latter approach. Leader self-sacrificial behavior (LSSB) is acts conducted by leaders who put aside their interests, rights, and privileges for the welfare of the organization (Choi and Yoon, 2005). Research has suggested that LSSB can signal to employees that organizational interests are very important and worth protecting even at the cost of self-interest (e.g., Choi and Mai-Dalton, 1998; Yang et al., 2022). This signal may likely enhance employees’ emphasis on organizational interests and make employees engage in less acts that are harmful to organizational interests. In this vein, we expect that LSSB has the potential to inhibit employees’ UPFB.

Theories of self and identity suggests that one’s self-referential description or identity has great effects on one’s behaviors and can be extended by including significant persons (e.g., Kelman, 1961; Banaji and Prentice, 1994; Leary and Tangney, 2003; van Knippenberg et al., 2004; Ashforth et al., 2016). This psychological merging of the self and another person (i.e., personal identification) leads people to internalize the values of that person, in much the same way as an extended self-concept to include a group (i.e., social identification) can prompt people to internalize the beliefs and norms of that group (Aron and McLaughlin-Volpe, 2001; Kark et al., 2003; Wang et al., 2017). Kelman (1961) pointed out that an influencing agent is usually the person who possesses attractive characteristics that can provide a satisfying self-definition. Given that conducting self-sacrificial behaviors for the sake of the organization reflects leaders’ personal qualities (Choi and Mai-Dalton, 1998; He et al., 2018), we expect that LSSB may prompt employees to identify with their leader and then internalize their leader’s pro-organizational values, inhibiting them from taking acts that may harm organizational interests. That is, identification with the leader may mediate the effect of LSSB on UPFB.

Moreover, although research suggests that the effects of LSSB are contingent on various factors (e.g., De Cremer, 2002; De Cremer et al., 2009) and can be explained by identity-based mechanisms (e.g., De Cremer and van Knippenberg, 2004; Li et al., 2016), not much is known about the boundary conditions under which LSSB more or less strongly affects employees’ identification with the leader and subsequent outcomes. To our knowledge, only one study has investigated this issue, finding that employees’ power distance weakened the positive impacts of LSSB on identification with the leader and citizenship behaviors (He et al., 2018). Given that LSSB is behaviors in which a leader engages to benefit the organization (Yang et al., 2022), we infer that for employees who perceive themselves to be organizational insiders (versus outsiders), when they observe their leader sacrificing his or her own interests to pursue organizational interests, their reactions may likely be more intense, as these employees attach more value to and are more sensitive to the organization-related issues (Choi et al., 2018). We thus propose that employees’ perceived insider status may function as a moderator of the relationships between LSSB, identification with the leader, and UPFB.

We examined our theoretical model using a sample of 236 Chinese working adults and adopting a time-lagged research design. Empirical results generally support our theorizing. By conducting this study, we contribute to the literature in several ways. First, as UPFB is a newly-proposed concept, the current investigation of this behavioral phenomenon is in its infancy (Cheng et al., 2022). By proving the negative influence of LSSB on UPFB, we not only enrich the research on the inhibitors of UPFB from the lens of leader behaviors, but also shed new light on the approaches of inhibiting UPFB (i.e., letting employees attach more importance to organizational interests). Second, premised on previous research on self and identity (van Knippenberg et al., 2004), this research proposed and examined the mediating role of identification with the leader, thus not only unpacking the potential mechanism via which UPFB can be inhibited by LSSB, but also extending the understanding of how to inhibit UPFB from the perspective of self and identity. Third, by simultaneously taking the employee–organization and leader–organization relationships into account and accordingly verifying the moderating role of perceived insider status in affecting LSSB effectiveness, we provide insight into the mechanism that explains when LSSB more strongly induces employees’ psychological and behavioral reactions.

## Theory and hypotheses

### Leader self-sacrificial behavior and unethical pro-family behavior

UPFB is unethical behaviors conducted by employees to intentionally benefit their family (Liu et al., 2020). Essential to this definition are two components that UPFB is undertaken with the intention to benefit the family but violates the widely accepted societal and organizational moral norms. UPFB is rather prevalent in the workplace and has high potential to damage organizational interests (Cheng et al., 2022). Typical examples of UPFB include spending work resources to cope with family-related issues when at work, taking family members to work to enjoy the firm benefits that were intended for employees, and so on (Liu et al., 2020). As UPFB is a newly-proposed type of workplace deviance, extant research on this behavioral phenomenon is rather limited. Up to now, scholars have found that some stressors in the family (e.g., family financial pressure; Liu et al., 2020) and work domains (e.g., workplace bullying; Yao et al., 2022) can promote UPFB and that family-related workplace support (e.g., family-supportive supervisor behavior; Cheng et al., 2022) can reduce UPFB. To better control this deviant behavior, more studies on the influencing factors of UPFB are warranted.

In this article, we study LSSB as a potential inhibitor of UPFB. LSSB refers to “any action in which a leader engages that is (a) a volitional behavior, (b) involving a cost to the agent (e.g., the loss of interests, privileges, or rights), and (c) for the benefit of others or the collective to which the self belongs” (Yang et al., 2022, p. 11). Put differently, LSSB is deliberately conducted by leaders for the pursuit of collective interests but at the expense of their own interests, such as assuming more work and forgoing rewards (Choi and Mai-Dalton, 1999; Choi and Yoon, 2005). In organizations, leaders play an important role in influencing employees’ value internalization and shaping their perceptions of certain behaviors (Li et al., 2016; Cheng et al., 2019). Self-sacrificial leaders give high priority to organizational goals and are willing to give up their interests to benefit the organization (De Cremer et al., 2009). They clearly express to employees the belief that organizational interests are important and should be put above personal interests. Affected by self-sacrificial leaders, employees may likely form pro-organizational values and become motivated to perform more pro-organizational behaviors and less actions that are detrimental to the organization (e.g., UPFB). In line with this thought, extant research has found that LSSB promotes employees’ organizational citizenship behaviors and restrain them from undertaking counterproductive work behaviors (e.g., De Cremer and van Knippenberg, 2004; Mostafa and Bottomley, 2020). Accordingly, we propose the following hypothesis:

H1: LSSB is negatively related to UPFB.

### The mediating role of identification with the leader

As a kind of personal identification, identification with the leader captures the extent to which the leader is included in the employee’s self-concept (Kelman, 1961; Kark et al., 2003; Ashforth et al., 2016). Prior research has suggested that leaders can profoundly affect employees’ self-concepts and thereby affect employees’ behaviors (Shamir et al., 1993; van Knippenberg et al., 2004). For instance, Li et al. (2017) found that a transformational leader’s individualized support and high-performance expectations enhanced employees’ identification with the leader, which in turn promoted their taking charge behavior. Ko et al. (2018) posited that personal identification is a vital conduit through which ethical leadership affected employees’ (un)ethical behaviors. Wang et al. (2017) found that servant leadership positively affected employees’ identification with the leader and then contributed to work-to-family positive spillover and subsequent work-family balance. Inspired by these studies, we infer that employees’ identification with the leader may mediate the link between LSSB and UPFB.

Specifically, according to personal identification theory, the admirable attributes of the target (e.g., morality, goals, and so on) are salient sources for one’s personal identification (Kelman, 1961; Ashforth et al., 2016). To benefit the collective, self-sacrificial leaders often volunteer risky actions and abandon their personal interests (Choi and Mai-Dalton, 1998; De Cremer and van Knippenberg, 2004). In their mind, the interests of the collective are paramount (Choi and Mai-Dalton, 1999). They have a strong sense of responsibility to ensure that their obligations and duties to the organization is fulfilled (De Cremer et al., 2009). As a result of all this, employees are very likely to form favorable perceptions of their self-sacrificial leaders (Mostafa and Bottomley, 2020). Research has indicated that self-sacrificial leaders are attributed more legitimacy and charisma and are generally perceived as moral and trustworthy (Halverson et al., 2004; Choi and Yoon, 2005; De Cremer and van Knippenberg, 2005). Thus, LSSB may likely enhance employees’ personal identification with the leader.

Furthermore, we expect that employees who identify with the self-sacrificial leader may engage in less UPFB. Identity and identification theorists have posited that one’s identification with another person can prompt one to adopt that person’s attributes (Ashforth et al., 2016). In other words, one’s identification with another person makes one tend to believe what that person believes and do what that person does so as to be like that person (Kelman, 1961). Following this logic, when employees identify with the self-sacrificial leader, they may likely internalize the pro-organizational values and behavioral mode of the leader. To become similar to the self-sacrificial leader, these employees may take organizational interests to heart and reduce the occurrence of behaviors that have the potential to cause damage to the organization (e.g., UPFB). Taken together, drawing on personal identification theory (van Knippenberg et al., 2004), we predict that leaders’ admirable attributes expressed by the form of LSSB will positively affect employees’ personal identification with the leader, which in turn inhibits employees from engaging in UPFB. Accordingly, we propose the following hypothesis:

H2: Identification with the leader mediates the relationship between LSSB and UPFB.

### The moderating role of perceived insider status

Although LSSB may enhance employees’ personal identification with their leader and subsequently inhibit their UPFB, we deem that the effects of LSSB may vary across employees. As a kind of pro-organizational behavior, LSSB mainly involves two relationship entities, namely the leader and the organization (Yang et al., 2022). Identification with the leader also mainly involves two relationship entities, namely the leader and the employee (Ashforth et al., 2016). Through the lens of relationship, it is possible that the employee–organization relationship may affect the relationship between LSSB and identification with the leader. Perceived insider status is an employee–organization relationship concept, reflecting the extent to which employees perceive themselves to be organizational insiders (Stamper and Masterson, 2002; Masterson and Stamper, 2003). Research has shown that perceived insider status moderates employees’ responses to organization-related issues (e.g., Li et al., 2014; Kim et al., 2019). We thus expect that perceived insider status may serve as a boundary of employees’ positive reactions to LSSB (i.e., enhanced identification with leader and reduced UPFB).

Perceived insider status is a perception regarding the relationship an employee has with his or her organization (Stamper and Masterson, 2002). Employees with high perceived insider status see themselves as the insiders of the organization and are usually more concerned about the things that happen to the organization than outsiders (Choi et al., 2018). This emphasis on organization-related things caused by employees’ high perceived insider status may affect insiders’ reactions of LSSB in two ways. First, when employees with high perceived insider status see their leaders enacting self-sacrificial behaviors for the sake of the organization, they may think that their leaders are also very concerned about the organization. This values similarity between the employee and the leader may likely enhance the employee’s identification with the leader (Kark et al., 2003) and then restrain the occurrence of UPFB. Second, when organizational insiders see their organizations receiving benefits from their leaders’ self-sacrificial behaviors, they may deem that their leaders care about them. This sense of leader caring may likely strengthen the identification with the leader (Ashforth et al., 2016) and prevent them from conducting UPFB. In contrast, employees with low perceived insider status care less about organization-related issues. As a result, when facing to LSSB, the reactions of such employees may be less intense than those of employees with high perceived insider status. That is, for organizational outsiders, although LSSB can still enhance their identification with the leader and then reduce their UPFB, these effects are merely based on employees’ perceptions of their leader’s admirable attributes included in behaviors. Accordingly, we propose the following hypothesis:

H3: Perceived insider status moderates the relationship between LSSB and identification with the leader such that the relationship is more positive when employees perceive themselves as organizational insiders than outsiders.

H4: Perceived insider status moderates the indirect effect of LSSB on UPFB via identification with the leader such that the indirect effect is more negative when employees perceive themselves as organizational insiders than outsiders.

**FIGURE 1:**
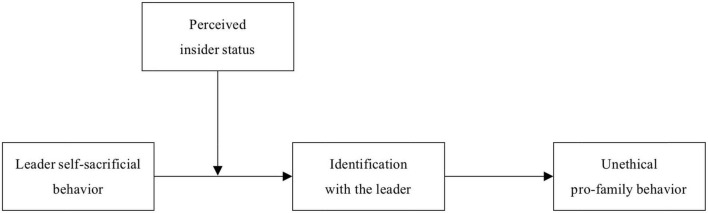
The research model.

Taken together, the theoretical model of our study can be depicted in Figure 1.

## Method

### Sample and procedure

We conducted a two-phase questionnaire survey to test our theoretical model, with a diverse sample recruited from Sojump, a Chinese online survey platform that has been widely used to obtain reliable data by organizational researchers (e.g., Chen et al., 2022; Wang et al., 2022). The industries involve retail, health service, catering, finance, construction, mechanical engineering, and so on. At the first phase, 412 participants were asked to report their LSSB, perceived insider status, identification with the leader, and some demographic information including gender, age, marital status, education level, and position level. Two weeks later, at the second phase, based on the questionnaire ID provided by the survey platform, 247 participants who participated in the first survey were successfully contacted and invited to report their social desirability and UPFB. The first-phase and second-phase data were matched according to the participant ID. We checked the data and filtered 11 invalid samples (e.g., the ones whose answers were self-contradictory). Among the 236 valid samples, 60.2% were female; the mean age were 31.61 years (SD = 5.63); 80.1% were married; 92.4% had a bachelor’s degree or above; 86.9% were general employees or front-line managers.

### Measures

Following the translation and back-translation procedure suggested by Brislin (1986), we translated the English scale collected from top-tier journals into Chinese versions. Unless stated otherwise, all items were measured with five-point Likert scales (1 = strongly disagree, 5 = strongly agree).

#### Leader self-sacrificial behavior

LSSB was measured with De Cremer and van Knippenberg’s (2004) five-item scale. A sample item is “My supervisor is willing to make personal sacrifices in the organizational interest.” The Cronbach’s α for this scale was 0.81.

#### Perceived insider status

We measured perceived insider status using Stamper and Masterson’s (2002) six-item scale. A sample item is “I feel very much a part of my work organization.” The Cronbach’s α for this scale was 0.84.

#### Identification with the leader

We used the seven-item scale developed by Shamir et al. (1998) to assess identification with the leader. A sample item is “My supervisor represents values that are important to me.” The Cronbach’s α for this scale was 0.88.

#### Unethical pro-family behavior

UPFB was assessed with Liu et al.’s (2020) seven-item scale. Participants were asked during the past month how frequently they engaged in behaviors like “To help my family, I took company assets/supplies home for family use.” UPFB was rated by a Likert-type scale ranging from 1 (“never”) to 5 (“all the time”). The Cronbach’s α for this scale was 0.88.

#### Control variables

As prior research has suggested that demographics may affect individuals’ unethical behavior (e.g., Liu et al., 2020; Cheng et al., 2022), we controlled for participants’ gender, age, marital status, education level, and position level. Gender (1 = male, 2 = female) and marital status (1 = married, 2 = single) were operated as binary variables. Education (1 = technical college or less, 2 = bachelor’s degree, 3 = master’s degree or above) and position (1 = general employee, 2 = front-line manager, 3 = middle or senior manager) were divided into three levels. Age was assessed in years. Besides, given that one’s social desirability may affect the report of morality-related items, we controlled for participants’ social desirability adopting the ten-item impression management subscale of social desirability developed by Steenkamp et al. (2010). A sample item is “When I was young, I sometimes stole things.” The Cronbach’s α for this scale was 0.83.

### Analytic strategy

First, we conducted a series of confirmatory factor analyses to test whether our measures captured distinctive constructs and whether the common method bias was a serious threat in this research. Second, we reported the means, standard deviations, and correlations of all variables. Third, to test the hypotheses, we adopted hierarchical regression analysis and the bootstrapping approach (5,000 repetitions). The aforementioned analyses were conducted by SPSS 26.0 and Mplus 8.3.

## Results

### Confirmatory factor analyses

Table 1 displays the confirmatory factor analysis results. As Table 1 shows, the four-factor model had better fit (χ^2^ = 396.57, df = 269, CFI = 0.95, TLI = 0.94, RMSEA = 0.05, SRMR = 0.05) than other models (i.e., three-factor, two-factor, and one-factor models), verifying the distinctiveness of our measures of LSSB, perceived insider status, identification with the leader, and UPFB. Moreover, following Podsakoff et al.’s (2003) recommendation, we used the unmeasured latent method factor approach to assess the issue of common method bias. The results showed that adding a common method factor did not result in significant improvements over the model fit indices (χ^2^ = 372.78, df = 263, CFI = 0.96, TLI = 0.95, RMSEA = 0.04, SRMR = 0.05), indicating that the common method bias was not a serious issue in our study (Dulac et al., 2008; Wang et al., 2019).

**TABLE 1:** Confirmatory factor analysis results.

Model	χ^2^	df	CFI	TLI	RMSEA	SRMR
Four-factor model: LSSB, PIS, IL, UPFB	396.57	269	0.95	0.94	0.05	0.05
Three-factor model: LSSB, PIS+IL, UPFB	659.98	272	0.85	0.83	0.08	0.08
Two-factor model: LSSB+PIS+IL, UPFB	971.61	274	0.72	0.70	0.10	0.11
One-factor model: LSSB+PIS+IL+UPFB	1,273.69	275	0.60	0.57	0.12	0.12

*N* = 236. LSSB, leader self-sacrificial behavior; PIS, perceived insider status; IL, identification with the leader; UPFB, unethical pro-family behavior. +Represents factors combined.

### Descriptive statistics

Table 2 shows the means, standard deviations, and correlations among variables. As expected, UPFB was negatively related to LSSB (*r* = –0.31, *p* < 0.01) and identification with the leader (*r* = –0.56, *p* < 0.01). LSSB was positively associated with identification with the leader (*r* = 0.29, *p* < 0.01).

**TABLE 2:** Means, standard deviations, and correlations.

Variable	*M*	SD	1	2	3	4	5	6	7	8	9
1. Gender	1.60	0.49									
2. Age	31.61	5.63	-0.16*								
3. Marital status	1.20	0.40	-0.16*	-0.25**							
4. Education level	2.01	0.40	-0.01	-0.21**	0.04						
5. Position level	1.52	0.72	-0.04	0.21**	-0.17**	-0.03					
6. Social desirability	2.50	0.46	-0.10	0.16*	0.14*	-0.08	-0.01				
7. LSSB	3.24	0.72	-0.11^†^	0.10	-0.02	0.08	0.02	0.01			
8. PIS	3.76	0.70	-0.05	0.08	-0.11	-0.01	0.02	-0.05	0.04		
9. IL	3.25	0.78	0.02	0.19*	-0.04	-0.07	-0.02	0.04	0.29**	0.49**	
10. UPFB	2.37	0.77	-0.11^†^	-0.09	0.02	0.01	0.03	-0.06	-0.31**	-0.27**	-0.56**

*N* = 236. LSSB, leader self-sacrificial behavior; PIS, perceived insider status; IL, identification with the leader; UPFB, unethical pro-family behavior.

^†^*p* < 0.10; *p < 0.05; **p < 0.01.

### Hypotheses testing

Table 3 presents the hierarchical regression analysis results. According to Table 3, LSSB negatively affected UPFB (*b* = –0.35, *p* < 0.01, Model 2) and positively affected identification with the leader (*b* = 0.31, *p* < 0.01, Model 6). Identification with the leader negatively affected UPFB (*b* = –0.55, *p* < 0.01, Model 3). When both LSSB and identification with the leader were included as the predictors of UPFB in Model 4, identification with the leader negatively affected UPFB (*b* = –0.50, *p* < 0.01), and so was LSSB (*b* = –0.19, *p* < 0.01), showing that identification with the leader partially mediated the negative relationship between LSSB and UPFB (Baron and Kenny, 1986). At the same time, we estimated the indirect effect of LSSB on UPFB via identification with the leader by adopting the bootstrapping approach. The results showed that this indirect effect was significant (indirect effect = –0.16, 95% CI = [–0.26, –0.08], excluding 0). Hence, H1 and H2 were supported.

**TABLE 3:** Hierarchical regression analysis results.

Variable	UPFB				IL			
	Model 1	Model 2	Model 3	Model 4	Model 5	Model 6	Model 7	Model 8
Constant	2.37**	2.37**	2.37**	2.37**	3.25**	3.25**	3.25**	3.25**
Gender	-0.22*	-0.27**	-0.17^†^	-0.20*	0.09	0.14	0.18*	0.18*
Age	-0.02^†^	-0.01	-0.01	0.01	0.03**	0.02	0.02*	0.02*
Marital status	-0.03	-0.04	-0.03	-0.03	0.02	0.03	0.12	0.15
Education level	-0.05	0.01	-0.08	-0.04	-0.06	-0.11	-0.12	-0.14
Position level	0.05	0.05	0.02	0.02	-0.07	-0.06	-0.06	-0.07
Social desirability	-0.09	-0.09	0.08	-0.08	0.02	0.02	0.06	0.08
LSSB		-0.35**		-0.19**		0.31**	0.30**	0.31**
PIS							0.54**	0.54**
LSSB × PIS								0.19*
IL			-0.55**	-0.50**				
*R* ^2^	0.03	0.13	0.33	0.36	0.04	0.12	0.35	0.35
Δ*R*^2^	0.03	0.10	0.30	0.23	0.04	0.08	0.23	0.02
Δ*F*	1.20	26.69**	102.44**	80.38**	1.68	20.80 **	79.67**	5.63*

*N* = 236. LSSB, leader self-sacrificial behavior; PIS, perceived insider status; IL, identification with the leader; UPFB, unethical pro-family behavior.

^†^*p* < 0.10; *p < 0.05; **p < 0.01.

**FIGURE 2:**
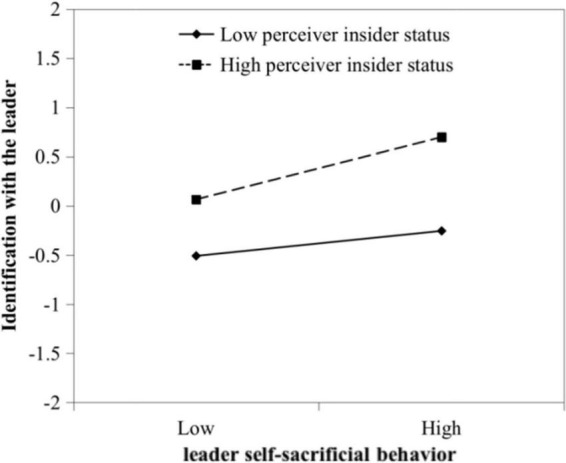
The moderating effect of perceived insider status on the relationship between leader self-sacrificial behavior and identification with the leader.

H3 predicted the moderating effect of perceived insider status on the relationship between LSSB and identification with the leader. According to Table 3, the interaction term labeled as “LSSB × PIS” positively influenced identification with the leader (*b* = 0.19, *p* < 0.05, Model 8). We used Aiken and West’s (1991) approach to plot the moderating effect (see Figure 2) and calculated the simple slopes. The results demonstrated that LSSB had a significant effect on insiders’ identification with the leader (slope = 0.44, *p* < 0.01) and that LSSB had a marginally significant effect on outsiders’ identification with the leader (slope = 0.18, *p* < 0.10). Taken together, H3 was supported.

To test H4, we followed Edwards and Lambert’s (2007) advice and estimated the indirect effects of LSSB on UPFB via identification with the leader at high and low levels of perceived insider status (i.e., one standard deviation above and below the mean of perceived insider status) by employing the bootstrapping approach. The results demonstrated the moderating effect of perceived insider status on the indirect effect of LSSB on UPFB through identification with the leader (indirect effect difference = –0.13, 95% CI = [–0.26, –0.01], excluding 0). Specifically, this indirect effect was significant for insiders (indirect effect = –0.22, 95% CI = [–0.35, –0.13], excluding 0) but was not significant for outsiders (indirect effect = –0.09, 95% CI = [–0.21, 0.01], including 0). Hence, H4 was supported.

## Discussion

Based on the perspective of personal identification and the diverse sample recruited from China, this study developed and examined a moderated mediation model to unpack why and when employees’ UPFB might be inhibited by LSSB. The results showed that LSSB negatively affected UPFB, in part due to employees’ identification with the leader. Additionally, employees’ perceived insider status could enhance the constructive effects of LSSB on employees: compared to outsiders, insiders would more strongly identify with their leader and then reduce UPFB when they experienced LSSB.

### Theoretical implications

Our study has several theoretical contributions. As noted at the outset, although increased academic attention has been devoted to employees’ UPFB (e.g., Li et al., 2022; Yao et al., 2022), how to inhibit this new type of workplace deviance is still understudied and less well understood. Inspired by Cheng et al.’s (2022) work, we explored the inhibitors of UPFB via the lens of leader behaviors. Meanwhile, different from prior studies that mainly explain the formation or inhibition of UPFB from the angle of employees’ family need (e.g., Liu et al., 2020; Cheng et al., 2022; Li et al., 2022), we tried to answer the question of how to inhibit UPFB from the angle of the importance of organizational interests. By verifying the inhibiting effect of LSSB, a kind of leader behaviors that attaches much importance to organizational interests, on UPFB, we not only respond to Liu et al.’s (2020) call for more studies on the influencing factors of UPFB, but also extend the understanding of how to restrain UPFB.

In addition, the negative relationship between LSSB and UPFB found in this study also adds to the literature on the outcomes of LSSB. While prior research has found that LSSB can significantly affect employees’ behaviors (e.g., cooperation; De Cremer et al., 2009; organizational citizenship behavior; He et al., 2018; taking charge; Li et al., 2016), the effects of LSSB on employees’ deviant behaviors have received little attention (Mostafa and Bottomley, 2020). This omission is somewhat regrettable, because employees’ deviant behaviors can also greatly affect the effective functioning of the organization (Dalal, 2005). Meanwhile, prior research on discretionary work behavior has found that the relationship between citizenship behavior and counterproductive behavior is not negative all the time (Dalal et al., 2009). Hence, the idea that the factor promoting pro-organizational behaviors (e.g., LSSB) must reduce destructive deviant behaviors needs to be empirically tested. By verifying the negative effect of LSSB on UPFB, this study extends the nomological network of LSSB.

Moreover, the present study sheds light on a specific psychological mechanism (i.e., personal identification) via which LSSB reduces employees’ UPFB. In a recent wave of leadership research, more and more scholars begin to consider the key role of identification in the process of leadership (e.g., Ashforth et al., 2016; Wang et al., 2017). This is not surprising, as prior multiple identification research has showed that factors associated with a given entity have stronger effects on identification with this entity and that low-order identities are more likely to have greater effects on one’s behavior than high-order ones (e.g., collective identity; Ashforth et al., 2008). We continued this emerging research stream and verified the mediating impact of identification with the leader, thus opening the black box of how LSSB inhibits UPFB to some extent. Meanwhile, beyond perspectives proposed in past research on the formation or inhibition of UPFB (e.g., social exchange perspective; Cheng et al., 2022; social cognitive perspective; Liu et al., 2020), we offered a new account based on a personal identification perspective, thus enriching the current understanding of how to control UPFB.

Finally, although prior research has widely investigated the boundaries of the impacts of LSSB on employees (Yang et al., 2022), little attention has been paid to the role of employee–organization relationship. This gap should be narrowed, as scholars have argued that employees’ perception and evaluation of organization-related issues (e.g., human resource management practices and leaders’ pro-organizational behaviors) are likely to be influence by their relationship with the organization (e.g., De Cremer et al., 2006; Liao et al., 2022). To our knowledge, among prior research on LSSB, only one research has tried to narrow this gap, which found that the impact of LSSB on employees’ trust in the leader was more positive for employees who strongly identified with their organization (De Cremer et al., 2006). Although perceived insider status and organizational identification belong to the category of employee–organization relationship, they are distinct (Stamper and Masterson, 2002). Hence, by verifying the moderating effects of perceived insider status on the links between LSSB and its outcomes, we extend the knowledge about the boundaries of the effects of LSSB.

### Managerial implications

In practical terms, our findings provide several implications for organizations to control and reduce UPFB. Specifically, we found that the leader’s self-sacrificial behaviors could enhance employees’ identification with the leader and then reduce the occurrence of UPFB. Thus, if managers or leaders want to restrain their subordinates’ UPFB, proactively performing self-sacrificial behaviors for the sake of the organization can be an effective strategy. Meanwhile, we advise that when selecting and promoting leaders, the organization should give more chances to leader candidates who give priority to organizational interests, even at the cost of their own interests. Pro-organizational values learning and training programs for leaders are also highly recommended, as such programs can help leaders shift their emphasis from the pursuit of their own interests to the pursuit of organizational interests. In sum, we deem that it is very necessary for the organization to take means to cultivate, maintain, and promote LSSB, especially during the post-pandemic period and global economic downtrend.

Additionally, we found that employees’ perceived insider status could strengthen the effects of LSSB on employees. To be specific, for employees perceiving themselves as organizational insiders, LSSB could effectively enhance their identification with the leader and then inhibit them from taking UPFB; but for employees perceiving themselves as organizational outsiders, the constructive effects of LSSB would be very limited. This is perhaps because organizational outsiders do not care about what their leader do for the organization or even may negatively attribute the leader’s self-sacrificial behaviors (e.g., impression management attribution). Thus, the organization should realize that LSSB is not the panacea for UPFB but has its functional boundaries. To ensure the effectiveness of LSSB, the organization is suggested to show more care and considerations to outsiders, such as assigning suitable tasks to them, listening their opinions, praising their work accomplishments, and so on.

### Limitations and future directions

Our study has some limitations that should be noted. First, because our samples were all from China, the single cultural context may limit the generalizability of our findings. Chinese society has a long history of assessing leaders on moral grounds and has a strong collectivistic culture (Hofstede, 2001; Farh et al., 2008). Leading by a virtuous and selfless person is usually appreciated by followers (Li et al., 2016). Consequently, employees in China may be more responsive to LSSB. We therefore advise future research to replicate our study using Western samples. Second, although we collected data at two time points with a 2-week interval, it is difficult for us make strong casual inferences, because the data were correlational in its essence. To address this limitation, future research is suggested to adopt experimental or longitudinal research designs so as to provide more compelling evidence for the casual relationships we proposed. Third, the number of samples reached in our study is somewhat insufficient. Although we initially contacted 412 participants, we failed to contact nearly 40 percent of them during the second-phase survey. We hope future research can learn some lessons regarding the multi-wave online survey from our study and increase the number of samples at the first phase to ensure the sufficient number of samples at the second phase. Last, we deem that future research may benefit from exploring other mediating mechanisms and boundary conditions. In terms of potential mediators, drawing on affective events theory (Weiss and Cropanzano, 1996), we infer that elevation, one type of other-praising moral emotions that arises due to witnessing another individual’s moral excellence (Greenbaum et al., 2020), may partially mediate the effect of LSSB on UPFB. In terms of potential moderators, as we initially analyzed in the managerial implications, employees’ attribution to LSSB (e.g., impression management attribution) may affect their perception of LSSB (Yang et al., 2022). If employees deem that the reason why the leader engages in self-sacrificial behaviors is to establish a favorable social image, their identification with the leader may less likely be induced.

## Conclusion

Drawing on personal identification theory, this study explored the questions of whether, why, and when UPFB could be inhibited by LSSB. Our analyses showed that LSSB can enhance employees’ identification with the leader, which in turn will prevent them from engaging in UPFB. Meanwhile, employees’ perceived insider status can strengthen the positive effect of LSSB on identification with the leader and the mediating effect of identification with the leader in the LSSB-UPFB relationship. Our findings enrich the knowledge of UPFB and LSSB and provide some practical suggestions that organizations can follow to prevent, control, and reduce employees’ UPFB.

## Data availability statement

The data that support the findings of this study are available from the corresponding author KC, chengken@zjut.edu.cn, upon reasonable request.

## Ethics statement

Ethical review and approval was not required for the study on human participants in accordance with the local legislation and institutional requirements. Written informed consent from the patients/participants or patients/participants legal guardian/next of kin was not required to participate in this study in accordance with the national legislation and the institutional requirements.

## Author contributions

KC, JH, and CH conducted conceptualization and the data collection and wrote the first draft of the manuscript. PH and YL performed data analyses. All authors contributed to the manuscript and approved the version to be published.
